# Sensing the Structural Differences in Cellulose from Apple and Bacterial Cell Wall Materials by Raman and FT-IR Spectroscopy

**DOI:** 10.3390/s110605543

**Published:** 2011-05-25

**Authors:** Monika Szymańska-Chargot, Justyna Cybulska, Artur Zdunek

**Affiliations:** Institute of Agrophysics, Polish Academy of Science, Doświadczalna 4, 20-290 Lublin, Poland; E-Mails: j.cybulska@ipan.lublin.pl (J.C.); a.zdunek@ipan.lublin.pl (A.Z.)

**Keywords:** Raman spectroscopy, FT-IR spectroscopy, cellulose, pectin, xyloglucan, *Gluconacetobacter xylinus*, crystallinity degree

## Abstract

Raman and Fourier Transform Infrared (FT-IR) spectroscopy was used for assessment of structural differences of celluloses of various origins. Investigated celluloses were: bacterial celluloses cultured in presence of pectin and/or xyloglucan, as well as commercial celluloses and cellulose extracted from apple parenchyma. FT-IR spectra were used to estimate of the I_β_ content, whereas Raman spectra were used to evaluate the degree of crystallinity of the cellulose. The crystallinity index (X_C_^RAMAN^%) varied from −25% for apple cellulose to 53% for microcrystalline commercial cellulose. Considering bacterial cellulose, addition of xyloglucan has an impact on the percentage content of cellulose I_β_. However, addition of only xyloglucan or only pectins to pure bacterial cellulose both resulted in a slight decrease of crystallinity. However, culturing bacterial cellulose in the presence of mixtures of xyloglucan and pectins results in an increase of crystallinity. The results confirmed that the higher degree of crystallinity, the broader the peak around 913 cm^−1^. Among all bacterial celluloses the bacterial cellulose cultured in presence of xyloglucan and pectin (BCPX) has the most similar structure to those observed in natural primary cell walls.

## 1. Introduction

Cellulose is the most abundant natural polymer. Each cellulose molecule is an unbranched polymer containing from 1,000 to 1 million D-glucose units, linked together with β-1,4 glycosidic bonds [1]. Celluloses from various sources are all the same at the molecular level, but they differ in their crystalline structures and the way that other biopolymers bind to them. Pure cellulose exists in several crystalline polymorphs with different packing arrangements, which are known as cellulose I, II, III and IV. Cellulose I is the most abundant naturally, cellulose II is also found in Nature produced by some bacteria or algae, but mostly, like cellulose III and IV, it can be prepared from cellulose I by chemical treatment [1,2].

Native cellulose is a mixture composed of two distinct crystalline phases, cellulose I_α_ and I_β_, which have the same conformations, but differ in crystal structure, having triclinic (I_α_) and monoclinic unit cells (I_β_). The proportion of I_α_ and I_β_ varies, depending on the source of the cellulose. Generally, cell walls of higher plants abound in cellulose I_β_, whereas cellulose I_α_ occurs mostly in algal or bacterial cell walls [2,3]. Celluloses I_α_ and I_β_ possess the same molecular building and O3-H–O5 intra-chain hydrogen bonding, but they have different O2-H–O6 inter-chain hydrogen bonding. In addition, cellulose I_β_ contains two chains in each monoclinic unit cell, while I_α_ contains one chain in the triclinic unit cell [4,5]. Celluloses I_α_ and I_β_ differ in the displacements of the sheets. The neighbouring sheets of cellulose I_α_ (consisting of identical chains with two alternating glucose conformers) are regularly displaced from each other in the same directions, whereas sheets of cellulose I_β_ (consisting of two conformationally distinct alternating sheets) are staggered [6–8]. Cellulose I_α_ is interconverted into I_β_ by bending during fibril formation [9].

Cell walls of plant tissue consist of two different phases: the microfibrillar and matrix phase [1]. The matrix phase is made up from a few polysaccharide components, namely pectins, hemicelluloses and also proteins and phenolics. The microfibrillar phase, which is distinguishable from the matrix phase by its high degree of crystallinity, consists of cellulose organized in microfibrils [1]. There is evidence of structural heterogeneity of each microfibril: a highly crystalline core surrounded by a less crystalline region and interrupted by amorphous forms of cellulose [1,10]. Physico-chemical behaviour, *i.e.*, accessibility for chemical derivatization, swelling and water binding and also mechanical properties of cell walls, which directly influence the textural properties of plant tissues, depends on the degree of cellulose crystallinity. The crystalline domains in cellulosic materials are of very limited size and are mixed with noncrystalline regions in fibrils, which predominantly determine their mechanical properties, such as tensile strength. The ratio of crystalline to total materials is termed the crystallinity index *X*_C_*%* and can be measured by a variety of methods, relying on different structural features, like X-ray diffraction, solid-state ^13^C-NMR or wide-angle X-ray scattering experiments [10–13]. The above mentioned methods can be supported by vibrational (infrared and Raman) spectroscopy, which are the simplest and the least time consuming methods of cellulose crystallinity index determination [11,13–17]. In this method crystallinity index is evaluated by measuring relative peak heights or areas beneath peaks [13,15,18,19]. An obvious disadvantage of this method is that it can give only relative crystallinity index values because the spectrum consists of contributions from both amorphous and crystalline regions.

The influence of the addition xyloglucan or pectin to bacterial media had been investigated previously and cellulose structural changes were observed [20–22]. Most of the researchers have concentrated on cellulose/xyloglucan or cellulose/pectin assemblies [23–28]. However, there is little work on the structural changes of cellulose produce by bacteria in the presence of pectin and/or xyloglucan in terms of FT-IR [20–22,24,29].

It has been shown previously that bacterial cellulose, enriched with pectin and xyloglucan, correctly simulates the structure and the chemical composition of the natural plant cell walls [30,31]. Hence, the bacterial cellulose is a convenient material for testing the interactions between polymers and effects of various food additives on the physical properties of cell walls. We have proposed that Raman and infrared spectroscopy would complete the knowledge on cellulose structure and polymer interaction of the model materials. Therefore in this paper, the influence of pectin, xyloglucan or both pectin and xyloglucan on structure changes of bacterial celluloses in terms of vibrational spectroscopy is studied.

The aim of this study was twofold: (i) to assess the structural differences between cellulose of various sources of origin, *i.e.*, commercial celluloses, bacterial cellulose and cellulose from apple cell walls with Raman and FT-IR spectroscopy; (ii) To investigate whether bacterial cellulose reflects the structure of natural cellulose from apple parenchyma.

## 2. Materials and Methods

### 2.1. Materials

In the experiment different types of cellulose were used: bacterial cellulose, commercial cellulose and cellulose extracted from apple tissue. For the isolation of the cellulose from apple tissue (*Malus domestica* cv. Ligol) fruits were used stored in cold room for two months directly after harvest. Apple pectin with a methylation degree at about 30% was purchased from Herbstreith and Fox (Neuenbürg, Germany). Xyloglucan from tamarind seeds (*Tamarindus indica* L.) was purchased from Megazyme (Bray, Ireland). Additionally, commercial microcrystalline celluloses were used: Avicel PH101 and PH302 (50 and 100 μm, alpha-cellulose obtained from wood pulp, FMC Biopolymer, Belgium), cellulose powder (20 μm, obtained from cotton linters, Sigma Aldrich) named further as Aldrich. All commercial celluloses were used without any further purification.

#### 2.1.1. Preparation of Bacterial Cellulose

Bacterial cellulose was obtained by purification of bacterial artificial cell walls produced in various media. Bacterial artificial cell walls materials were produced using the protocols described by Cybulska *et al.* [30,31]. Briefly, lyophilised culture of *Gluconacetobacter xylinus* (strain NRRL B-759 (ATCC 10245, NCIB 8034) from the USDA National Center of Agricultural Research) was cultured in liquid Hestrin Schramm culture medium (HS) containing glucose 2%, bactopeptone 0.5%, yeast extract 0.5%, disodium phosphate 0.27%, citric acid 0.115%, pH was adjusted to 5.0 with 5 M NaOH [32]. The temperature of incubation was 28 °C. After 24 h Petri dishes with agar HS medium were inoculated and incubated at 28 °C. The inoculum was prepared by transferring a single *G. xylinus* colony from HS agar medium into a 250 mL Erlenmeyer flask containing 50 mL of liquid HS medium. The strain was incubated under a constant temperature of 28 °C and with delicate stirring to ensure aeration [31]. *Gluconacetobacter xylinus* is a simple aerobic Gram-negative bacteria which has an ability to synthesize a large quantity of high-quality cellulose organized as twisting ribbons of microfibrillar bundles [33]. The artificial cell walls were grown on the top of the medium. Depending on the medium composition, different artificial cell walls were obtained:

- BC—bacterial cellulose;
- BCX—bacterial cellulose embedded in xyloglucan, obtained by adding xyloglucan from tamarind seeds to the medium up to 5 g L^−1^;
- BCP—bacterial cellulose embedded in pectin, obtained by adding apple pectin to the medium up to 5 g L^−1^ and 12.5 mM calcium chloride;
- BCPX—bacterial cellulose with pectin and xyloglucan created by adding apple pectin (2.5 g L^−1^), xyloglucan (2.5 g L^−1^) and 6 mM calcium chloride to the culture medium.

The temperature of incubation was 28 °C for BC, BCX, BCP and BCPX. Thin films of BC, BCX, BCP and BCPX were collected 7 days after inoculation. Films of pure bacterial cellulose (BC) were washed many times in distilled water, and in the case of materials containing pectin and xyloglucan (BCP, BCX and BCPX), in 12.5 mM solution of calcium chloride. The materials were stored in 0.02% sodium azide solution at 4 °C prior to testing to prevent degradation.

In order to test the influence of matrix polysaccharides during production of cellulose microfibrils in culture medium on cellulose crystallinity the microfibrils were isolated from artificial cell walls. In order to remove culture medium and bacterial cells which might be trapped in material and cause fluorescence during recording of Raman spectra artificial cell walls (BC, BCP, BCX, BCPX) were washed three times in 0.1 M HCl at 85 °C and three times in 1M NaOH at 80 °C for 30 min each bath. Bacterial cellulose was washed several times in boiling deionised water (100 °C). This procedure is believed not to change the structure and mechanical properties of cellulose [31,34,35] and is also used to solubilise pectins and hemicelluloses [29]. After this purification there was no evidence of bands originated from xyloglucan and pectin in the Raman and infrared spectra of bacterial celluloses. Also, we have not observed any bands corresponding to peptidoglycan from bacterial cell walls which would be visible in the 1,200–900 cm^−1^ region [36,37]. However in general, the main two sugar-amine components of the bacterial peptidoglycan (N-acetylglucosamine and N-acetylmuramic acid) can be measured or determined by FT-IR or Raman spectroscopy as well as the Gram negative polysaccharide layers/leaflets of the cell wall [36,37].

#### 2.1.2. Extraction of Apple Cellulose

Cellulose from apple tissue was obtained during sequential extraction. Briefly, apple cell wall material was isolated using modified phenol-buffer method as proposed by Renard [38]. Frozen apple slices were homogenised in a cool buffer simulating the ionic conditions in apple juice (1.2 mM CaCl_2_, 2.0 mM MgCl_2_, 0.5 g L^−1^ KCl, 60 mg L^−1^ ascorbic acid, 4 g L^−1^ apple acid, 1 g L^−1^ sodium disulfite supplemented to pH 3.5 with 5 M NaOH) with Triton 100 (2 g L^−1^) and 1-octanol (4 mL). The suspension was then filtered under reduced pressure and washed in 60% water solution of acetone. The resultant paste was blended with phenol at a volumetric ratio of 1:4 and left for one hour at room temperature. Next, the blend was dissolved in the buffer and filtered. The material was washed successively in 70% and 96% ethanol, and finally in acetone. The sample was vacuum dried. Further, the obtained cell walls were purified by the method proposed by Redgwell *et al.* with some modifications [39]. Cell walls (45 mg) was stirred in 0,1 M CDTA (pH 6.5, 4.5 mL) at 25 °C for 6 h. Then it was filtered and the residue diluted in 0.05 M CDTA (pH 6.5, 4.5 mL) and stirred at 25 °C for 2 h and again filtered. Then residue was stirred in 0.05M Na_2_CO_3_ (8 mL) with addition of 20 mM NaBH_4_ for approx. 20 h at 1 °C, filtered, and again stirred for 2 h at 20 °C. After filtration, the residue (depectinated cell walls) was stirred sequentially in 0.5 M, 1 M and 4 M of KOH (with a addition of 20 mM NaBH_4_ each) 6 mL for 2 h at 20 °C every time. Finally all cellulose from apple tissue was rinsed several times in deionised water. At all stages of filtration 65 G filter paper was used.

### 2.2. Methods

#### 2.2.1. Raman Spectroscopy

The cellulosic powders and bacterial cellulose films (BC, BCX, BCP, BCPX) were applied on a microscope glass. Raman spectra were collected on a DXR Raman Microscope (Thermo Scientific, Waltham, MA, USA) with laser 532 nm and maximum power 10 mW. The spectra were recorded over the range 3,500–150 cm^−1^ using an operating spectral resolution of 1.9285 cm^−1^ of Raman shift. Spectra were taken with 2 s exposure and 10 mW laser power output. For each material, 15 samples were examined under the same conditions. For each sample, 64 scans were averaged. Then for a given material, final average spectrum was calculated. These spectra were normalized to 1.0 at 2,900 cm^−1^.

#### 2.2.2. FT-IR Spectroscopy

FT-IR spectra were collected on a Nicolet 6700 FT-IR (Thermo Scientific, Waltham, MA, USA). The Smart *iTR* ATR sampling accessory was used. The commercial celluloses and apple cellulose were applied on ATR as powders, whereas purified celluloses produced by bacteria were dried and placed on ATR as membranes. In order to check whether the form of the sample has any influence on the spectra, the two forms were compared for bacterial cellulose. No differences in band shape and intensity were visible, and there were no differences between spectra obtained from membranes and powders of bacterial cellulose. The spectra were collected over the range 4,000–650 cm^−1^. For each material, five samples under the same conditions were examined. For each sample, 200 scans were averaged with a spectral resolution of 4 cm^−1^. Then for a given material, a final average spectrum was calculated. These spectra were normalized to 1.0 at 1,136 cm^−1^ (COH stretching vibration). Baseline corrections were obtained on Omnic Software (Thermo Scientific).

#### 2.2.3. %I_β_ Determination

In the FT-IR spectra of cellulose, bands of hydrogen bonding vibrations are present in the 700–800 cm^−1^ range. It has been observed that two peaks around 750 and 710 cm^−1^ are characteristic for I_α_ and I_β_ allomorphs, respectively [5,20,40]. The relative proportion of cellulose I_β_ to I_α_ allomorph could be calculated by integrating the absorption bands near 710 and 750 cm^−1^ and the percentage of I_β_ could be obtained by Equation (1) [5,18,20,40–42]:

(1) $$\%{\text{I}}_{\beta }\;=\;\frac{{\text{A}}_{710}}{{\text{A}}_{710}\;+\;{\text{A}}_{750}}$$

where A_710_ and A_750_ are integrated intensities of the bands around 710 and 750 cm^−1^.

#### 2.2.4. Cellulose Crystallinity Degree Determination

Schenzel *et al.* have shown that the intensity of peaks at 1,462 and 1,481 cm^−1^, which correspond to CH_2_ bending, relates to crystalline/amorphous proportions in a cellulosic sample [13]. The higher the peak at 1,481 cm^−1^, the higher the cellulose crystallinity degree is too. In the case when amorphous cellulose predominates over crystalline one, there is only evidence of a broad peak at 1,462 cm^−1^ and some deconvolution must be made. On this basis one can estimate the crystalline index by counting a relative percentage amount of crystalline fraction in a cellulosic sample. Thus, degree of crystallinity could be calculated by Equation (2) [13]:

(2) $$\%{\text{X}}_{\text{C}}^{\text{RAMAN}}\;=\;\frac{{\text{I}}_{1481}}{{\text{I}}_{1481}\;+\;{\text{I}}_{1462}}\times 100\%$$

were I_1462_ and I_1481_ represent the Raman intensities of the particular bands at 1,462 and 1,481 cm^−1^, respectively.

## 3. Results and Discussion

### 3.1. Raman Spectra

**Figure 1.**
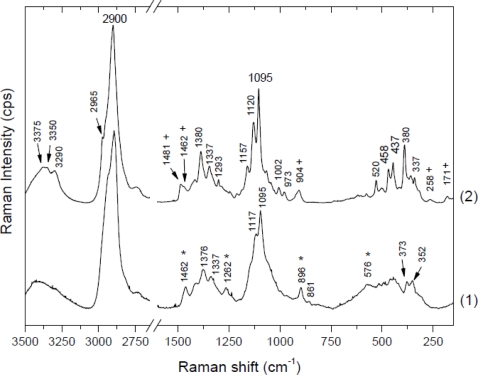
Raman spectra of apple (1) and microcrystalline (2) cellulose in the range 3,500–2600 and 1,600–150 cm^−1^. There are marked peaks characteristic for amorphous (*) and crystalline (+) celluloses.

Raman spectra can be divided into two regions. The region below 1,600 cm^−1^ (especially below 700 cm^−1^) is most sensitive to the conformation of the cellulose backbone, whereas the region above 2,700 cm^−1^ is more sensitive to hydrogen bonding [43,44]. Figure 1 presents Raman spectra of cellulose I for the 150–1,650 cm^−1^spectral range. The spectra are for microcrystalline celluloses (cellulose powder, Aldrich) and for celluloses from apple parenchyma as an example of different proportions of highly crystalline and more amorphous cellulose, respectively. Comparing these spectra, broadening and loss of resolution of apple cellulose could be observed. This would suggest that microcrystalline cellulose contains a higher percentage of crystalline cellulose than apple cellulose. Another difference in the Raman spectra between apple and microcrystalline celluloses lies in band location and these are collected in Table 1.

It must be remembered that cellulose possesses many more vibrational degrees of freedom than is observed in Raman spectra, therefore some modes are probably degenerated. In the region below 1,500 cm^−1^ there are particular assignments resulting from internal motions of methylene groups.

Skeletal bending modes of CCC, COC, OCC, OCO are dominant in the 150–550 cm^−1^ region. Additionally, it involves methane bending (CCH, COH) and movement of CC, CO groups within the glucopyranose ring units. Between 550 and 800 cm^−1^ bands are weak, widely spaced or do not occur at all. However, there are some differences in the 150–800 cm^−1^ region between microcrystalline and apple cellulose. The most visible distinction, apart from worse resolution, is a lack of peaks around 171 cm^−1^ and 258 cm^−1^, which probably indicates COH bending [15,43].

The 800–1,180 cm^−1^ region is dominated by CC and CO stretching motions and some amounts of HCC, HCO bendings. Significant differences appear in the intensity of the peak around 913 cm^−1^, which are probably related to cellulose crystallinity and particularly to the size of crystallites. Atalla and Wiley have observed that the larger crystallites and therefore more homogeneous cellulose (peaks are better resolved and narrower) are associated with a lower intensity of this peak [43]. In Figure 1, this peak for apple cellulose is narrower and shifted to lower frequencies (896 cm^−1^) than for microcrystalline cellulose (904 cm^−1^). This would indicate a higher amount of disorder in the apple cellulose.

In the 1,180–1,270 cm^−1^ region bending of HCC, HCO, HCH and COH is predominant; in the 1,270–1,350 cm^−1^ one bending of HCC, HCO; and in the 1,350–1,430 cm^−1^ one bending of COH. There are only small differences between the spectra of apple and microcrystalline cellulose and they mostly concern the resolution of the peaks.

Significant differences appear in the range 1,430 and 1,500 cm^−1^, which indicated differences in HCH bending. Microcrystalline cellulose contains two peaks at 1,462 and 1,481 cm^−1^, whereas apple cellulose has only one broad peak around 1,462 cm^−1^. It was observed that a higher peak at 1,481 cm^−1^ corresponds with a higher degree of crystallinity [45]. The lack of this peak for apple cellulose could confirm the amorphous properties of this material. In Figure 1 one can observe that some bands in the apple cellulose spectrum, for example, 576, 896, 1,262, 1,462 cm^−1^, are similar to those obtained by Schenzel *et al.* for cellulose II polymorph [15]. Davis and Harris have noticed that an extraction procedure using 4 M KOH results in conversion of cellulose I into cellulose II and also production of amorphous cellulose. In Nature only a few bacterial strains could produce the cellulose of modification II, therefore the changes of apple cellulose probably are a result of the preparation process [46].

**Figure 2.**
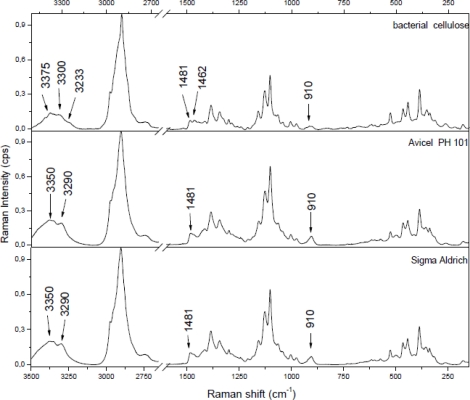
Comparison of bacterial cellulose and two microcrystalline celluloses (Aldrich and Avicel PH101). Raman spectra in the range 3,500–2,600 and 1,600–150 cm^−1^.

Between 1,500 and 2,500 cm^−1^ there is no evidence of bands in the Raman spectra of cellulose. Above 2,500 cm^−1^ two bands occur: a very intensive one around 2,900 cm^−1^, which indicates mostly CH stretching vibrations and a very broad one between 3,200 and 3,500 cm^−1^ corresponding to OH stretching vibrations. In this region there are no clear differences between apple cellulose and microcrystalline cellulose, except for the lower resolution in the case of apple cellulose. Comparison of bacterial cellulose and microcrystalline cellulose (Figure 2) shows only differences in three regions:

1. around 910 cm^−1^—this peak is broad and less intensive in the bacterial cellulose case; as it has been mentioned this peak is probably related with the lateral size of crystallites; addition of pectin and/or xyloglucan changes the size of cellulose crystallites;
2. peaks at 1,462 and 1,481 cm^−1^—different proportions of these peaks indicate different degrees of crystallinity in the samples;
3. 3,100–3,500 cm^−1^ region—differencee in band assignment between bacterial cellulose and microcrystalline cellulose. In this region different proportions of cellulose allomorphs (I_α_ and I_β_) are revealed.

### 3.2. Content of Cellulose I_α_ and I_β_

**Figure 3.**
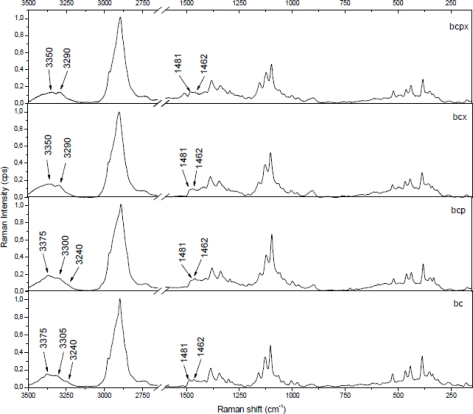
Comparison of bacterial celluloses cultured in presence of pectin (BCP), xyloglucan (BCX), both (BCPX) or neither (BC) Raman spectra in the 3,500–2,600 and 1,600–150 cm^−1^ range.

The 3,100–3,500 cm^−1^ region corresponds to O-H stretching vibrations. As it can be seen from Figure 2, there are characteristic lines at 3,233, 3,300 and 3,375 cm^−1^ for bacterial cellulose and at 3,350 and 3,290 cm^−1^ for both microcrystalline celluloses. This diversity indicates different hydrogen bonding for microcrystalline and bacterial celluloses. Atalla and Wiley have observed different bond patterns for celluloses obtained from *Cladophora glomerata*, which is known to be pure cellulose I_β_ and from *Valonia ventricosa*-rich in cellulose I_α_ [11,47]. They have noticed in this region that I_β_ posses a higher frequency shoulder (around 3,400 cm^−1^), whilst Raman spectra of I_α_ show a line peak at 3,240 cm^−1^, which did not occur in spectra of I_β_. Bacterial cellulose is known to be rich in I_α_, whereas microcrystalline cellulose, obtained from fibrous higher plants is rather rich in I_β_. However native cellulose is a mixture of these two allomorphs of cellulose I and the proportion of I_α_ to I_β_ in plant tissue varies depending on its origin [7]. An influence of xyloglucan and pectin on cellulose formation in terms of Raman spectra was also investigated (Figure 3). Bacterial celluloses vary in the 3,100–3,500 cm^−1^ region. For BC and BCP, patterns are the same and indicate a high content of cellulose I_α_. However spectra for BCX and BCPX are rather similar for this obtained for cellulose I_β_. To confirm this observation, FT-IR spectra of microcrystalline celluloses and BC, BCP, BCX, BCPX were collected.

**Figure 4.**
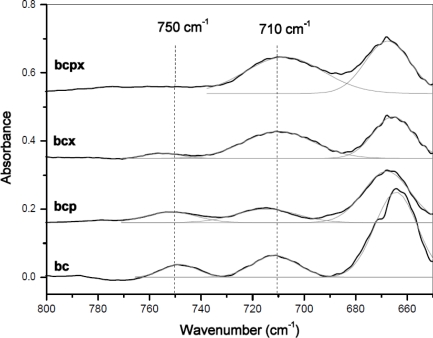
Comparison of FTIR spectra of bacterial celluloses cultured in presence of pectin (BCP), xyloglucan (BCX), both pectin and xyloglucan (BCPX) or neither (BC) in the range 800–650 cm^−1^.

In the case of BCPX and all microcrystalline celluloses actually there is a very weak absorption band around 750 cm^−1^, whilst the band around 710 cm^−1^ is slightly shifted to 700 cm^−1^. There is no evidence of a band at 750 cm^−1^ in the FT-IR spectra of cellulose from cotton linters, which is practically pure cellulose I_β_ [13]. Despite the weak absorption at 750 cm^−1^ in the FT-IR an estimation of %I_β_ can be made. For all spectra, a Gaussian function was fitted under the peaks around 750 and 710 cm^−1^ and then the area under the peaks was collected (Figure 4).

**Figure 5.**
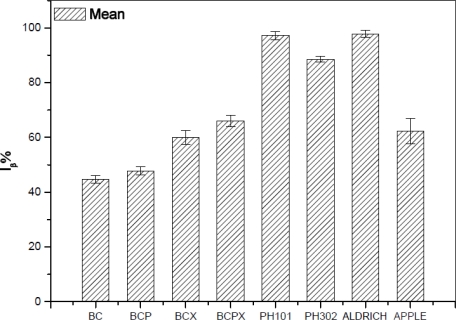
The percentage contents of cellulose I_β_ for celluloses from different origin.

As it was expected after Raman spectra analysis, FT-IR confirmed that the most I_α_-rich cellulose was in the BC and BCP materials (Figure 5). In terms of Raman spectra microcrystalline celluloses as well as BCX and BCPX are rich in cellulose I_β_, although IR spectra of BCX indicate the presence of the low intensity peak at 750 cm^−1^. After calculation of %I_β_, we obtained the lowest value for BC and BCP (45% and 49%, respectively, Table 2).

Our results for BC are nearly in the range of 60–70% of I_α_ previously found for *Acetobacter xylinus* (synonym *Gluconacetobacter xylinus*) ATCC 53524 [20,25,48,49]. The previous studies revealed that addition of pectins (BCP) has no significant influence on the structure of the formed cellulose and %I_β_ is 30%, which is comparable with pure bacterial cellulose (BC) [45]. The result obtained by us is higher, however it is still similar to the %I_β_ of BC, which confirms this hypothesis. In presence of xyloglucan alone or with addition of pectins we observed increasing %I_β_, up to 60 and 66%, respectively. It was reported before that in cellulose produced by *Acetobacter xylinum* in the presence of water-soluble hemicelluloses (xyloglucan) the proportions of I_β_/I_α_ are increasing and the size of crystallites is decreasing [14,20,42]. The percentage content of %I_β_ in BCX and BCPX is close to the results obtained for apple cellulose (62%), which confirms that bacterial cellulose with addition of xyloglucan or xyloglucan and pectin could be used as a model cell wall material. Previous atomic force microscope (AFM) studies have shown that the most suitable as the model cell wall is BCPX, because apart from its similar composition it also has similar nano-topographical futures [30]. The result of the present experiment confirms that BCPX has the most similar cellulose structure for cellulose obtained from higher plants. In the case of commercial celluloses microcrystalline cellulose Avicel PH302 has an %I_β_ of 88%, whilst for Avicel PH101 the result is close to that of the Aldrich one, containing practically pure I_β_ in cotton linters (97%, Figure 5).

The role of pectin and hemicelluloses (xyloglucan) is to bind to cellulose microfibrils in cell walls. Xyloglucan coats the cellulose microfibrils, attaches to their surface as well as between microfibrils, limiting their aggregation. Pectin is believed to form an independent network, which works as plasticizer and water binding agent [29,50]. Absorption of xyloglucan on cellulose microfibrils is practically the same in the presence of low concentrations of pectin as in their absence. On the other hand, in the case of the presence of pectin, an increase of xyloglucan concentration causes a decrease of pectin absorption onto cellulose. The cellulose-pectin interaction seems to be weaker than the cellulose-xyloglucan interaction [29].

### 3.3. Crystallinity of Cellulose

The highest calculated degree of crystallinity obtained for microcrystalline cellulose is 53%. For apple cellulose estimation of crystallinity is difficult due to the low intensity of the peak at 1,481 cm^−1^ however a rough estimation suggests a value of only about 25%. This confirms that apple cellulose is mostly amorphous [51]. The intensity of peaks was estimated after applying a deconvolution function and a summary of results is presented in Table 2.

Cellulose powders, Avicel PH101 and PH302, have similar crystallinity degrees, around 51–53%. The results are generally in good correspondence with those acquired by Schenzel *et al.*, only the degree of crystallinity of Avicel PH302 obtained in this experiment is much lower [13,45]. Among bacterial celluloses the most crystalline one is BCPX. Addition of pectin or xyloglucan separately causes a decrease of degree of crystallinity. However, the addition of a mixture of pectins and xyloglucan did not affect the degree of crystallinity much, an even caused a slight increase. The low value of the degree crystallinity of apple cellulose (25%) could be the result firstly of KOH treatment in the extraction procedure [46] and secondly the overall high pectin content in apple parenchyma [52].

**Figure 6.**
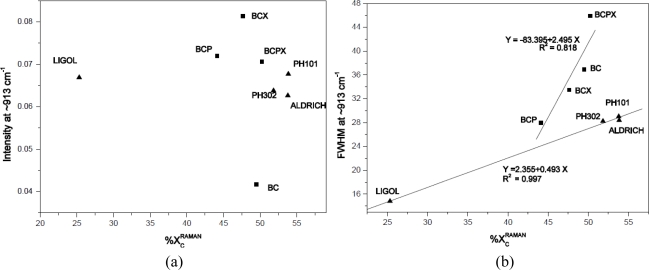
**(a).** The peak intensity around 913 cm^−1^ plotted against degree of crystallinity calculated by Equation 2 for celluloses of various origins. (**b**). The peak full width at half maximum (FWHM) around 913 cm^−1^ plotted against degree of crystallinity calculated by Equation (2) for celluloses of various origins.

Crystallinity degree results were compared with intensity and FWHM (Full Width at Half Maximum) of the peak around 913 cm^−1^. Atalla and Wiley have concluded that the higher and narrower the peak around 913 cm^−1^ is, the lower the degree of crystallinity is and the amount of disorder is higher [47]. Atalla and Wiley based their conclusions on the observation that there is a connection between band shape and intensity at 913 cm^−1^ of investigated ramie, cotton, bacterial, algal and amorphous cellulose and the degree of crystallinity of cellulose determined by ^13^C-NMR investigations [47]. In this experiment the intensities and FWHM of the ∼913 cm^−1^ peak were obtained by fitting a Gaussian function for each spectrum obtained and then average values were calculated. In Figure 6(a,b), the ∼913 cm^−1^ peak intensity and FWHM were plotted against the degree of crystallinity evaluated from Raman spectra, respectively.

As it was expected the width of the ∼913 cm^−1^ peak (FWHM) is increasing with increasing crystallinity, whereas the degree of crystallinity doesn’t show any dependence on the peak intensity. Interestingly, the average values for particular celluloses are grouped into two clusters containing all bacterial or microcrystalline cellulose. For both regression lines a positive correlation was found with coefficient of determination 0.818 (bacterial celluloses) and 0.997 (microcrystalline celluloses). Despite the slight decrease of peak ∼913 cm^−1^ intensity with degree of crystallinity observed [Figure 6(a)], the correlation is very weak. However, in Figure 6(a) clusters are also visible for bacterial or microcrystalline celluloses. It seems that the results for apple cellulose should be grouped with microcrystalline cellulose due to the fact, that microcrystalline celluloses and apple cellulose were investigated as powders whereas bacterial celluloses were investigated in the form of membranes. The size of cellulose crystallites for Aldrich, Avicel PH101 and PH302 are 20, 50 and 100 μm, respectively. As one can see from Figure 6(a,b) the point location is related negatively with the scale of fragmentation.

**Figure 7.**
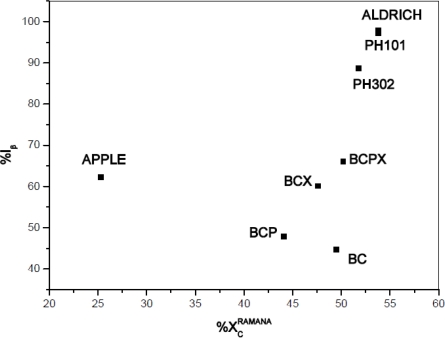
Dependence of cellulose degree of crystallinity from percentage content of cellulose I_β_.

The degree of crystallinity should also be affected by the structure of cellulose allomorphs, therefore it should depend on the I_β_/I_α_ composition of cellulose. The degrees of crystallinity were compared with %I_β_ content for the investigated celluloses (Figure 7). It seems that content of cellulose I_β_ correlates positively with degree of crystallinity, however for apple the result again differs clearly.

The role of pectins and xyloglucan additives are still under investigation. Non-cellulosic cell wall polysaccharides definitely interfere with the way cellulose microfibrils aggregate [20]. The addition of pectins practically didn’t influence the allomorphic structure and proportions of I_β_/I_α_ for BCP which remained the same as for BC. Formation of established cellulose allomorphs has an impact on the size of microfibrils: I_α_ tends to crystallize in the wider forms, whereas the I_β_-rich cellulose forms microfibrils of smaller size [20]. However, pectins and cellulose seem not to interact with each other [49], in compositions with low concentration of xyloglucan absorption of pectins onto microfibrils tends to increase. It was also observed that a higher amount of xyloglucan prevent pectins from absorbing onto microfibrils [29]. The findings of Zykwinska *et al.* suggest that pectins play a double role in the complex cellulose/xyloglucan/pectin systems. In the presence of high concentrations of xyloglucan pectins are loosely attached to cellulose microfibrils, whereas in compositions with low abundance in xyloglucan, the main function of pectins is to bind the gap between microfibrils [29].

## 4. Conclusions

Raman and infrared spectroscopy have been proven to be convenient ways of cellulose structure assessment in terms of crystallinity and allomorph structure. Various Raman bands can be used for successful and fast determination of crystallinity. FT-IR spectroscopy was found to be a sensitive method to estimate changes of the cellulose structure as a result addition of non-cellulosic polysaccharides. Changes of cellulose structure under the influence of pectins and xyloglucan were also investigated. These investigations have evidenced that addition of pectin and xyloglucan to cultured bacteria medium changes the structure of the cellulose produced and its degree of crystallinity. Bacterial cellulose enriched by both pectin and xyloglucan (BCPX) has a structure similar to those observed in natural primary cell walls. Therefore BCPX could be used as a model cell wall for testing polymer interactions and mechanical properties. However, it must be remembered that such models don’t include the effects of other hemicelluloses and lignins on cellulose and the next step of investigations of model cell walls should be incorporation of other non-celulosic plant cell wall polysaccharides.

Since cellulose naturally occurring in cell walls aggregates in the presence of different proportions of pectin and xyloglucan, further studies should be directed to structure investigations of cellulose obtained from different plants. Such research would allow for determination of how cellulose allomorphic structure and degree of crystallinity influences the mechanical properties of cell walls.

## Figures and Tables

**Table 1.** Summary of FT Raman frequencies and band assignments of crystalline (microcrystalline cellulose powder Sigma Aldrich) and amorphous (from apple cell walls) celluloses (s—strong, w—weak).

**Crystalline (cm^−1^)**	**(Amorphous cm^−1^)**	**Band assignment**^(1)^
171 w	-	COH methane bending
258 w	-	
337 w	-	CCC, COC, OCC, OCO skeletal bending, CCH, COH methane bending, movement of CC, CO groups within the glucopyranose ring units
356 s	352 w	
380 s	373 w	
437 s	low resolution	
458 s	low resolution	
520 s	576	
904 w	896 w	HCC, HCO bending
973 w	-	HCH bending
1,095 s	1,095 s	COC stretching symmetric
1,120 s	1,117 s	
1,157 s	-	CC, CO stretching asymmetric
-	1,262 w	HCH (twisting), HCC, HOC, COH (rocking) bending
1,293 w	-	HCH (wagging), HCC, HOC, COH (rocking) bending
1,337 s	1,337 w	
1,380 s	1,376 w	HCH, HCC, HOC, COH bending
1,462 w	1,462 w	HCH scissoring bending
1,481 w		

(1) Band assignments after Attalla and Wiley [15] and Schenzel and Fischer [43].

**Table 2.** Calculated values of cellulose crystallinity, percentage content of cellulose I_β_, FWHM (full width at half maximum) and intensity of Raman peak around 913 cm^−1^ for bacterial, microcrystalline and apple celluloses. In parenthesis standard deviation.

	**%X**_C_^RAMAN^	**%I**_β_	**FWHM ∼913 cm**^−1^	**INT∼913 cm**^−1^
BC	**49.5** (3.2)	**44.7** (1.5)	**36.9** (3.4)	**0.042** (0.012)
BCP	**44.1** (5.1)	**47.8** (1.5)	**27.9** (9.0)	**0.072** (0.045)
BCX	**47.6** (2.7)	**60.1** (2.5)	**33.5** (2.8)	**0.081** (0.025)
BCPX	**50.2** (1.4)	**66.0** (2.2)	**45.9** (9.9)	**0.071** (0.018)
PH101	**53.8** (1.4)	**97.2** (1.5)	**28.4** (2.9)	**0.068** (0.015)
PH302	**51.8** (2.4)	**88.6** (1.1)	**28.2** (2.2)	**0.064** (0.010)
Aldrich	**53.8** (1.4)	**97.8** (1.3)	**29.0** (2.5)	**0.063** (0.012)
Apple	**25.3** (1.8)	**62.3** (4.7)	**14.8** (0.8)	**0.067** (0.007)

## Abbreviations

BC: artificial cell walls of bacterial cellulose;
BCP: artificial cell walls of bacterial cellulose with pectin added during fermentation process;
BCX: artificial cell walls of bacterial cellulose with xyloglucan added during fermentation process;
BCPX: artificial cell walls of bacterial cellulose with pectin and xyloglucan added during fermentation process;
Aldrich: cellulose powder from Sigma Aldrich;
PH101 and PH302: cellulose powders from FMC Biopolymer;
FT-IR: Fourier transform infrared spectroscopy.
